# Supplementary figures and images for: Enhancement of osmotic stress tolerance in soybean seed germination by bacterial bioactive extracts
Source: PLoS One. 2023 Oct 12;18(10):e0292855. doi: 10.1371/journal.pone.0292855 (PMC10569584; doi:10.1371/journal.pone.0292855)

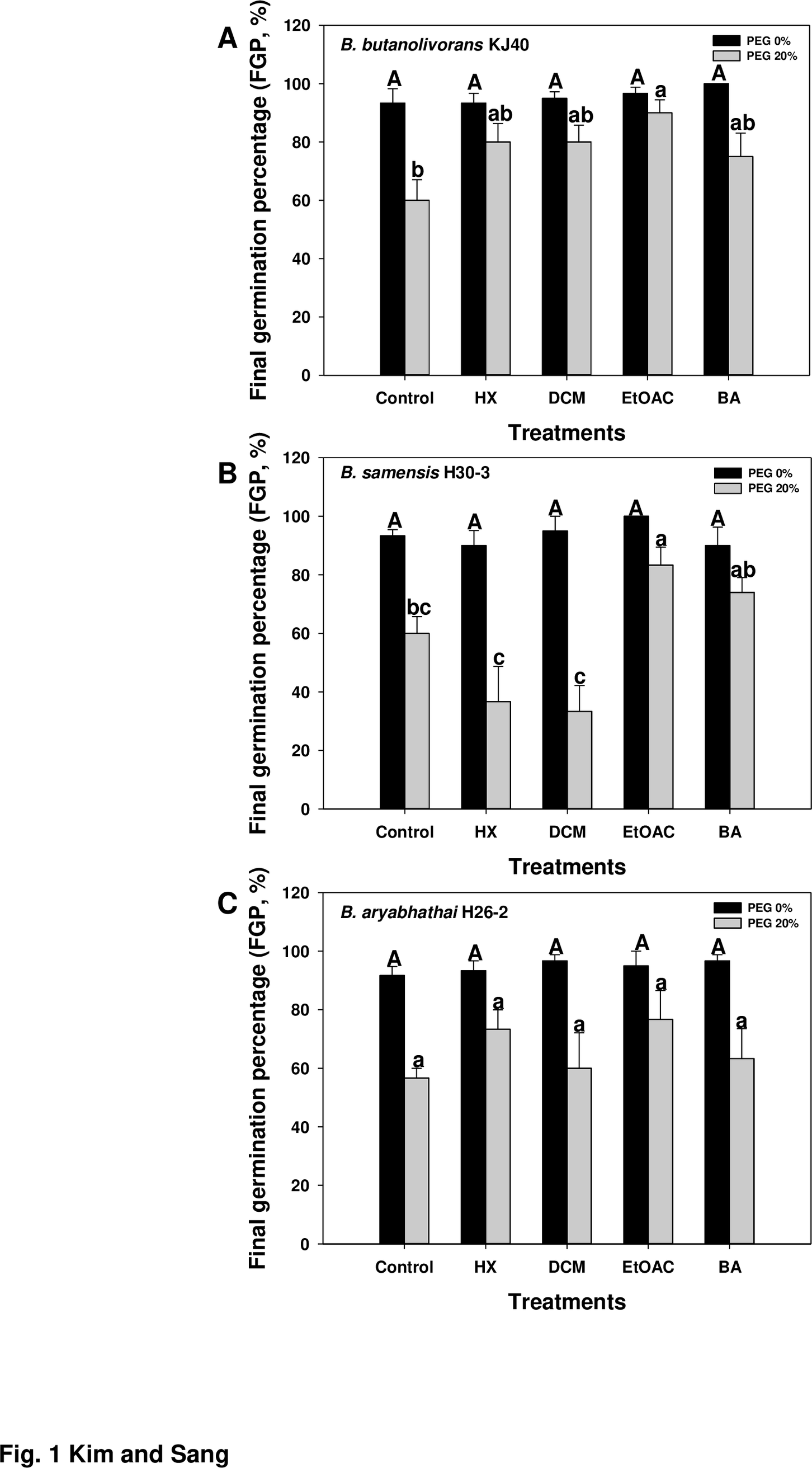

Supplement: S1 Fig — Final germination percentage of KJ40 (A), H30-3 (B), and H26-2 (C) under 0% or 20% PEG. Data presented as means + standard error; small letters on the bar mean significant difference (n=6, statistical significance assessed by the LSD test). (TIF) [file pone.0292855.s001.tif]
