# Supplementary material for: Enhancement of osmotic stress tolerance in soybean seed germination by bacterial bioactive extracts
Source: PLoS One. 2023 Oct 12;18(10):e0292855. doi: 10.1371/journal.pone.0292855 (PMC10569584; doi:10.1371/journal.pone.0292855)
Supplement: S1 Table — (DOCX) [file pone.0292855.s002.docx]

| Strains | Df | Sum Sq | Mean Sq | *F* value | *Pr*(>*F*) |
| --- | --- | --- | --- | --- | --- |
| KJ40 | 1 | 4411 | 4411 | 27 | 2.95E-06 |
| H30-3 | 1 | 11726 | 11726 | 39.56 | 8.37E-08 |
| H26-2 | 1 | 10848 | 10848 | 39.49 | 5.58E-08 |

**Supplemental table 1**. Anova analysis for Final germination percentage compared to conditions including 0% and 20% PEG.
