# Supplementary material for: Enhancement of osmotic stress tolerance in soybean seed germination by bacterial bioactive extracts
Source: PLoS One. 2023 Oct 12;18(10):e0292855. doi: 10.1371/journal.pone.0292855 (PMC10569584; doi:10.1371/journal.pone.0292855)
Supplement: Attachment — Submitted filename: Letter_25.7.23.docx [file pone.0292855.s003.docx]

**Academic Editor’s Comments**

**Enhancement of osmotic stress tolerance in soybean seed germination by bacterial bioactive extracts**

The research article has been written well by the authors. However, some points are there which must be cleared. The Introduction part should be framed in respect of the current research's objectives. Material and Methods should be complete. Results part should be more qualitative. Conclusions should be clear. The overall English grammar of the manuscript needs to be improved. The manuscript can be accepted after these changes and those suggested by the reviewers.
