# Supplementary material for: Enhancement of osmotic stress tolerance in soybean seed germination by bacterial bioactive extracts
Source: PLoS One. 2023 Oct 12;18(10):e0292855. doi: 10.1371/journal.pone.0292855 (PMC10569584; doi:10.1371/journal.pone.0292855)
Supplement: Attachment — Submitted filename: Response to Reviewers.docx [file pone.0292855.s004.docx]

Reviewer #1

Line 46-48: The future perspective is not very much clear, I suggest to rewrite the concluding remark sentence and “may show” should be replaced.

Response: We have made a change to the sentence (lines 47-48)

Line 54: This study is not country specific so I recommend please remove “including the United States, Brazil, Argentina, and China”.

Response: We have removed the phrase “including the United States, Brazil, Argentina, and China” (line 54)

Line 63: please remove and here in the sentence “physiological and biochemical”, and it should be replaced with coma (,).

Response: We have replaced the word “and” with a coma (,) (line 62)

Line 66-67: Water stress and drought have same meaning and only one word should be used here. Please re write this sentence as follows “Soybeans are highly sensitive to water stress, and it can severely impact their growth and production”.

Response: Thank you for the comment. We have made a change to the sentence (line 65)

Line 70-72: “During the plant’s maturation, drought stress can also cause wilting, leaf yellowing, and premature leaf drop owing to increased water loss through transpiration in response to elevated temperatures” should be rewritten as During the plant’s maturation, drought stress also cause wilting, leaf yellowing, and premature leaf drop owing to increased water loss through transpiration in response to elevated temperatures”.

Response: Thank you for your feedback. We have made the change to the sentence as you suggested. (lines 77-80)

Line 129-131: “Glycine max ‘Daewon’ was surface-sterilized by in 2% sodium hypochlorite,” Remove by in this sentence.

Response: We removed the word “by” from the sentence (line 141)

Some sentences are very long and I suggest need to break it into shorter ones.

Response: We restructured several sentences as follows:

∙ Lines 198-215 have been divided into lines 211-220 and 221-229

∙ Lines 223-239 have been split into lines 237-248 and 249-256

∙ Lines 254-285 have been reorganized into lines 271-293 and 294-305

I recommend to cite these references about ABA and drought stress in the Discussion section such as:
Batool et al., 2019; Partial and full root-zone drought stresses account for differentiate root-sourced signal and yield formation in primitive wheat”. Plant Methods
Gui et al., 2020; Differentiate effects of non-hydraulic and hydraulic root signaling on yield and water use efficiency in diploid and tetraploid wheat under drought stress”. Environmental and Experimental Botany
Lv et al., 2019; Comparative response to drought in primitive and modern wheat: a cue on domestication. Planta

Response: We have incorporated an additional sentence along with the provided references in the discussion section (lines 400-402)

Line 340: “with a decrease in oxidative stress Our findings indicated that MDA content was higher in seeds” Please add a full stop here between the two sentences.

Response: We have separated the two sentences (lines 359-360)

Line 367-368: “The GA stimulates seed germination, plant growth, and development by promoting hydrolytic enzyme production and inducing softening of the seed cover [54]”. And should be replaced with by i.e. “production and inducing”.

Response: We have replaced the sentence (lines 387)

Please elaborate Conclusion section more precisely.

Response: We have made change the paragraph (line 428-440)

Reviewer #2

1. The short term HAT is not appropriate in many places, such as Abstract, figures. It is better just use the full-name with only three words "hours after treatment".

Response: We have replaced the words (line 39, 44, 239-243, 245-247 250-253, 273-274, 276-284, 286-287, 289, 291-292, 294-297, 299-300, 302, 327, 397, 400, and 408-409. figure 1, 2, 3, 4, and 6)

2. line 174, "as described by xx", should be the author.

Response: We have added author’s name in place of “xx” (line 187)

3. page 9, what is the meaning for "two experiments", it might be better as "two treatments"?

Response: We pooled the data from two sets of experiments based on a statistical analysis of the homogeneity of variance.

4. In Table 2, a and b in the footnote is the same as a,b,c for statistic test, so it is suggested to change one of them.

Response: We have made a change in the footnote (Table 2)

5. In the Results part form lines 254 to line 285, it is better to divided it into two to three paragraph, to clearly show the GA and ABA pathway genes.

Response: We have divided two paragraph (lines 271-293 and 294-305)

6. Styles of some references should be carefully checked.

Response: We have checked the references

Reviewer #3

The introduction is too generic. It is not clear from the introduction why these parameters and genes were analysed. Several pieces of information which should be in the introduction are given in the discussion, for example lines 365-376, lines 381-386. Moreover, the discussion contains a repetition of results in too many details. Therefore, the discussion is too descriptive and the results are not properly discussed.

Response: We have added some sentences (lines 69-77)

Lines 121-123 – it is not clear how the extraction was performed.

Response: We have replaced the extraction methods (lines 128-132)

Line 130 – it is not clear how much of the extract was used since 100 μg/ml is not informative. What is 100 μg?

Response: We have added the amount of extraction (lines 133-137)

Line 135 – it is unclear what the authors meant by fraction and concentration selection.

Response: We have replaced the unclear phrase “fraction and concentration selection” with a more precise explanation (lines 147-149)

Line 136 – what light conditions were used?

Response: We have added light conditions (line 149)

The description of results should be more qualitative.

Response: We restructured several sentences as follows:

∙ Lines 198-215 have been divided into lines 211-220 and 221-229

∙ Lines 223-239 have been split into lines 237-248 and 249-256

∙ Lines 254-285 have been reorganized into lines 271-293 and 294-305

We have adjusted the references number due to the inclusion of additional sentences in the manuscript.

Lines 72, 74, 77, 80, 82, 85, 87-88, 92-93, 95, 97, 99, 104, 107-108, 111, 114, 127, 145, 156, 162, 167, 183, 187, 339, 351, 360, 369, 374, 380, 385, 387, 393, 395, 400, 402, 404, 407, and 415-416; Table 1
